# Accuracy of Modified Budin Views for the Femoral Neck Anteversion at Different Hip Abduction Angles: An Experimental Study on Dry Bones

**DOI:** 10.3390/diagnostics16081238

**Published:** 2026-04-21

**Authors:** Murat Yuncu, Emre Mucahit Kartal, Sacide Efsun Urger, Levent Sarıkcıoglu, Serkan Gurcan, Ozkan Kose

**Affiliations:** 1 Department of Orthopedics and Traumatology, Elmalı State Hospital, 07716 Antalya, Turkey; m-yuncu@yandex.com; 2 Department of Orthopedics and Traumatology, Antalya City Hospital, 07070 Antalya, Turkey; 3 Department of Radiology, Antalya City Hospital, 07070 Antalya, Turkey; 4 Department of Anatomy, Akdeniz University Medical Faculty, 07050 Antalya, Turkey; 5 Gurcan Orthopaedics Clinic, 34365 Istanbul, Turkey; 6 Department of Orthopedics and Traumatology, University of Health Sciences, Antalya Education and Research Hospital, 07100 Antalya, Turkey

**Keywords:** femoral neck, femur/anatomy and histology, hip joint/diagnostic imaging, radiography, tomography, X-ray computed, reproducibility of results, cadaver

## Abstract

**Background/Objectives:** The modified Budin radiographic technique is a practical alternative to CT for measuring femoral neck anteversion (FNA); however, the impact of hip abduction angle on its accuracy remains unclear. This experimental study examined how varying abduction angles affect agreement between modified Budin measurements and CT. **Methods**: Twenty-seven dry adult femora underwent CT scanning, and FNA was measured using a validated three-slice superimposition method as the reference standard. Modified Budin radiographs were obtained at 20°, 30°, and 40° of femoral abduction. Two orthopedic surgeons independently measured FNA on all images twice, with at least 15 days between measurements. Intra- and interobserver reliability were assessed using the intraclass correlation coefficient (ICC). Mean values per femur were analyzed. Agreement with CT was evaluated using Pearson correlation, Bland–Altman analysis, and absolute error comparisons across abduction angles. **Results:** Reliability was excellent across all modalities (ICC, 0.982–0.998). Mean CT-derived FNA was 10.0° ± 8.5°, compared with 9.1° ± 8.0° at 20°, 8.3° ± 7.8° at 30°, and 7.8° ± 7.5° at 40° of abduction (*p* < 0.001). Correlation with CT was strong at all positions, but systematic underestimation increased with abduction angle. Among the tested positions, 20° abduction showed the smallest bias, the narrowest limits of agreement, and the lowest absolute error. **Conclusions**: Hip abduction angle significantly influences the accuracy of the modified Budin view. Under controlled experimental conditions, 20° abduction provided the closest agreement with CT among the tested positions. These findings suggest that lower abduction angles may improve geometric accuracy, although clinical feasibility and performance must be confirmed in vivo before routine clinical application can be recommended.

## 1. Introduction

Femoral neck anteversion (FNA) is the angular relationship between the femoral neck axis and the distal femoral condylar axis and is an important morphologic parameter in hip biomechanics and orthopedic decision-making. Accurate assessment of FNA is relevant in deformity correction, total hip arthroplasty, hip preservation procedures, and the evaluation of rotational abnormalities of the femur [1,2,3]. Because measurement errors may affect preoperative planning and postoperative interpretation, reliable assessment of femoral version remains clinically important [2,3].

Computed tomography (CT) is widely regarded as the reference standard for measuring femoral anteversion because it allows precise visualization of the femur and accurate definition of both the femoral neck and condylar axes [4,5]. However, its routine use may be limited by higher radiation exposure, greater cost, and reduced availability compared with plain radiography. In addition, CT-based assessment often requires post-processing and technical expertise [6]. Therefore, although CT remains the preferred reference method, a simpler low-radiation radiographic alternative may still be desirable in selected clinical settings.

**Figure 1:**
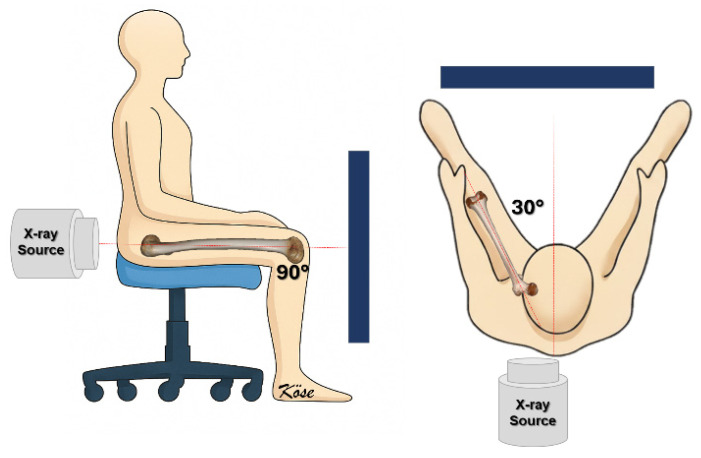
Schematic illustration of the modified Budin view used to measure femoral neck anteversion. (**Left**): The subject is seated with the hip and knee flexed to 90°, and the thigh resting on the chair, while the X-ray beam is centered on the hip joint and directed horizontally toward the detector placed behind the limb. (**Right**): Axial view showing the femur positioned in 30° hip abduction.

To address these limitations, various radiographic methods have been developed to measure femoral anteversion using plain films, ranging from complex biplanar techniques to simpler single-view projections [7,8,9]. One of the earliest and most distinct direct radiographic techniques for assessing femoral anteversion was described by Budin and Chandler in 1957 [10]. In their method, the patient was seated with the knees flexed to 90° to align the femoral axis with the central X-ray beam. Although this technique was effective in infants, it was technically demanding and yielded suboptimal image quality in adults and in children older than 6 years. To overcome these limitations and to enable measurement of femoral stem version in adult patients undergoing total hip arthroplasty, Lee et al. introduced the “modified Budin method” in 2013 [11]. In this modification, the patient is positioned sitting with the hip and knee each flexed to 90°, with an additional 30° of hip abduction, and a posteroanterior projection is used instead of the original anteroposterior projection to obtain a clearer axial view of the femur (Figure 1). Femoral stem version is then determined as the angle between the stem–neck axis and the posterior intercondylar line. This technique has the advantages of using standard radiographic equipment, involving lower radiation exposure than CT, and being relatively fast and straightforward to perform in routine clinical practice [12].

The accuracy of the modified Budin method is likely influenced by the degree of hip abduction used during imaging. In most published descriptions, a hip abduction angle of 30° has been adopted, largely on empirical grounds, as a compromise between patient comfort and an acceptable geometric projection of the femoral neck. Very low abduction angles, such as 0° or 10°, have not been historically favored because, in the seated modified Budin position, the trunk, abdomen, or contralateral limb may interfere with beam projection or detector placement, making image acquisition more difficult. As a result, greater abduction angles have traditionally been preferred to obtain a clearer axial-like projection. Moreover, some authors have even reported acquiring images with the hip abducted to 45° [13]. From a geometric standpoint, however, changes in hip abduction alter the spatial orientation of the femoral neck relative to both the distal femoral axis and the X-ray beam, thereby affecting the projected relationship between these structures and, consequently, the measured anteversion angle. Excessive abduction may lead to foreshortening or elongation of the femoral neck on radiographs, distorting the visualization of its true axis and introducing systematic error. In contrast, lower abduction angles may yield a projection that more closely approximates the true three-dimensional femoral neck anteversion [14].

To our knowledge, no study has systematically compared modified Budin measurements obtained at different abduction angles against CT-derived anteversion. Therefore, the aim of this study was to compare femoral neck anteversion measured on modified Budin views acquired at 20°, 30°, and 40° of abduction with CT-based measurements. We hypothesized that lower abduction angles would provide closer agreement with CT and reduce systematic measurement error.

## 2. Materials and Methods

### 2.1. Study Design and Specimens

This experimental study was conducted on dry human femora obtained from the osteology collection of the Anatomy Laboratory at Akdeniz University Medical Faculty. All available femora (*n*:159) were first inspected macroscopically, and fractured, deformed, or incomplete specimens were excluded. The sex and age of the specimens were unknown; however, all femora displayed closed physes, indicating skeletal maturity. The study protocol was reviewed and approved by the institutional ethics committee (Approval date/issue: 8 January 2026/160.2026), and all procedures were conducted in accordance with relevant guidelines, regulations, and the Declaration of Helsinki. The study used anonymized dry femoral specimens from the departmental osteology collection; therefore, no individual-level informed consent was required, and the committee waived the requirement.

### 2.2. Sample Size Calculation

Sample size calculation was based on the variability reported in the method-comparison study by Boissonneault et al., in which the limits of agreement (LoA) between CT and the modified Budin view were ±6.2°, corresponding to a standard deviation (SD) of approximately 3.1° for CT-radiograph differences [12]. Using this SD in a paired-sample design and assuming a clinically acceptable mean difference (Δ) of 2° between CT and radiographic measurements, the required sample size was calculated with the formula *n* = [(*Z_α_*_/2_ + *Z_β_*) × *SD*_diff_/Δ]^2^. For α = 0.05 (two-sided) and 90% power, the resulting sample size was 25 femurs. To account for potential case exclusion due to technical issues during imaging and thereby avoid loss of statistical power, 27 eligible cadaveric dry femora were randomly selected from the collection and included in this study.

### 2.3. CT Imaging and Measurements

**Figure 2:**
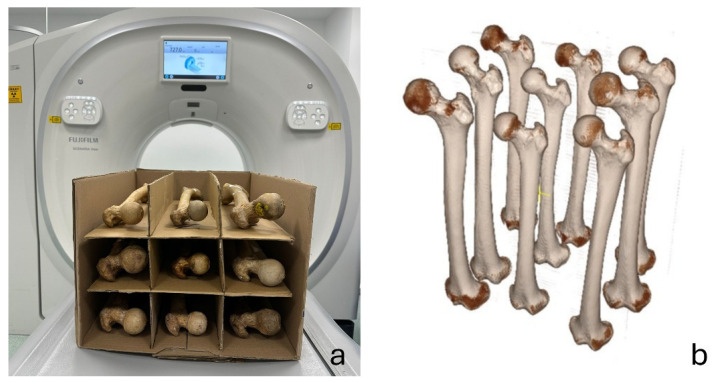
CT scanning setup. (**a**) Custom-made cardboard box with nine separate compartments used to position the dry femora for simultaneous CT acquisition in the multidetector CT scanner. (**b**) Three-dimensional volume-rendered reconstruction of the scanned femora generated from the CT data.

Femoral neck anteversion (FNA) was measured on computed tomography (CT) images and served as the reference standard. The femora were scanned using a multidetector CT scanner (Scenaria View, Fujifilm, Lexington, MA, USA). The specimens were placed in a custom cardboard box with nine separate compartments, and nine femora were scanned simultaneously in each acquisition (Figure 2). The scanning protocol included a field of view (FOV) of 50 cm, tube voltage of 120 kV, tube current of 92 mAs, and a slice thickness of 1 mm.

**Figure 3:**
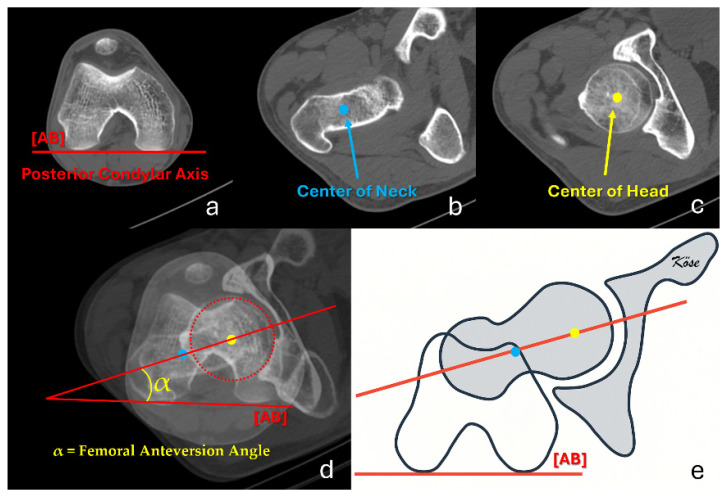
CT-based measurement of femoral neck anteversion (FNA). (**a**) Distal axial CT slice showing the posterior femoral condylar axis [AB]. (**b**) Axial slice at the level of the widest portion of the femoral neck with the neck center (blue) identified. (**c**) Axial slice at the level of the largest diameter of the femoral head with the head center (yellow) identified. (**d**) Superimposition of the three slices demonstrating the femoral head–neck axis (red line through yellow and blue points) and the posterior femoral condylar axis [AB]; the angle α between these two lines represents the FNA. (**e**) Schematic illustration of the same measurement technique.

All images were exported in DICOM format and transferred to a dedicated CT workstation (Sectra IDS 7, Sectra AB, Linköping, Sweden) for analysis. The most commonly used measurement methods described in the literature were adopted for FNA assessment [15]. For each femur, three axial CT slices were selected: (1) a slice showing the largest diameter of the femoral head, (2) a slice demonstrating the widest portion of the femoral neck, and (3) a distal slice showing the femoral condyles at their greatest width. On the proximal images, the center of the femoral head and the midpoint of the femoral neck were identified, and the line connecting these two points was defined as the femoral head–neck axis [16]. On the distal image, a line connecting the most posterior points of the medial and lateral femoral condyles was drawn and defined as the posterior femoral condylar axis [17]. These three images were then superimposed using the workstation software, and the angle between the femoral head–neck axis and the posterior femoral condylar axis was recorded as the FNA for each specimen (Figure 3).

### 2.4. Radiographic Imaging and Measurements

**Figure 4:**
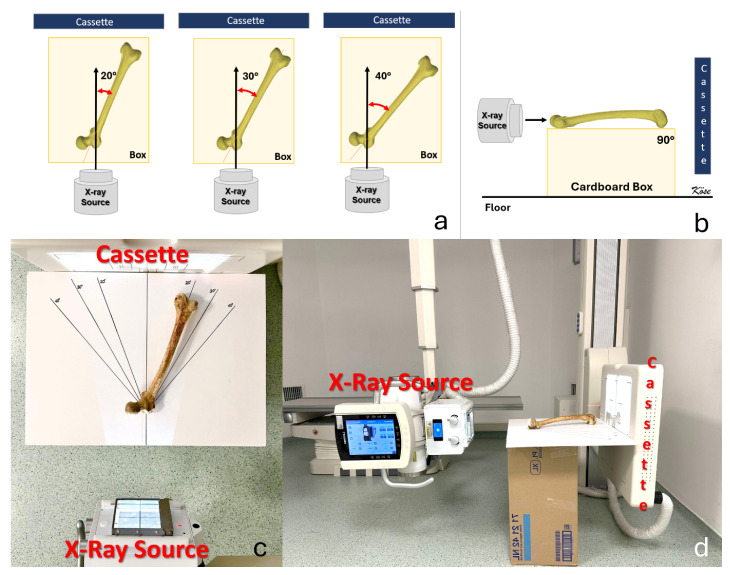
Experimental radiographic setup for modified Budin views at different hip abduction angles. (**a**) Schematic illustration of the femur positioned at 20°, 30°, and 40° of abduction relative to the midline, mimicking different hip abduction angles. (**b**) Lateral schematic view showing the femur aligned horizontally on a cardboard support with the X-ray beam directed perpendicular to the shaft to obtain the modified Budin projection. (**c**) Dry femur placed on a radiolucent surface with pre-drawn angular lines representing 20°, 30°, and 40° relative to the midline. (**d**) Clinical radiography setup demonstrating the position of the X-ray source and cassette during acquisition of the modified Budin radiographs.

**Figure 5:**
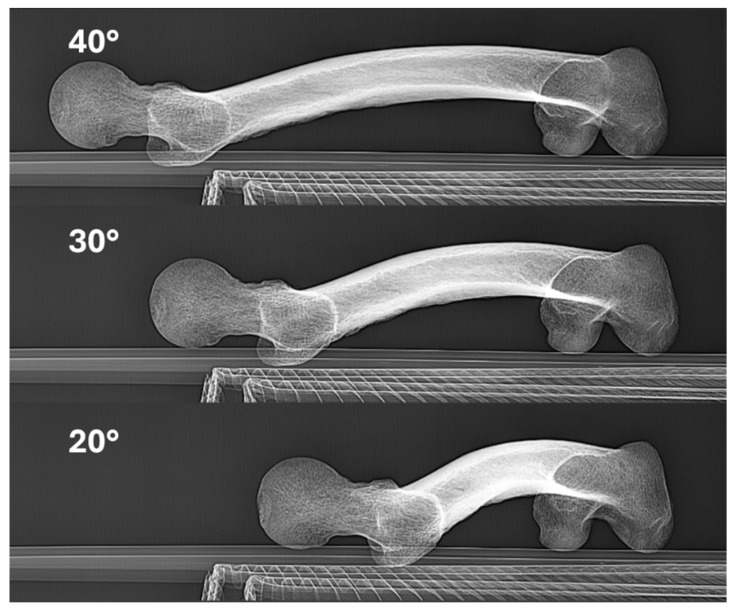
Representative modified Budin radiographs at different abduction angles. Posteroanterior radiographs of the same dry femur obtained with the shaft positioned at 40°, 30°, and 20° of abduction relative to the midline, illustrating the three modified Budin views used for femoral neck anteversion measurements in the study.

**Figure 6:**
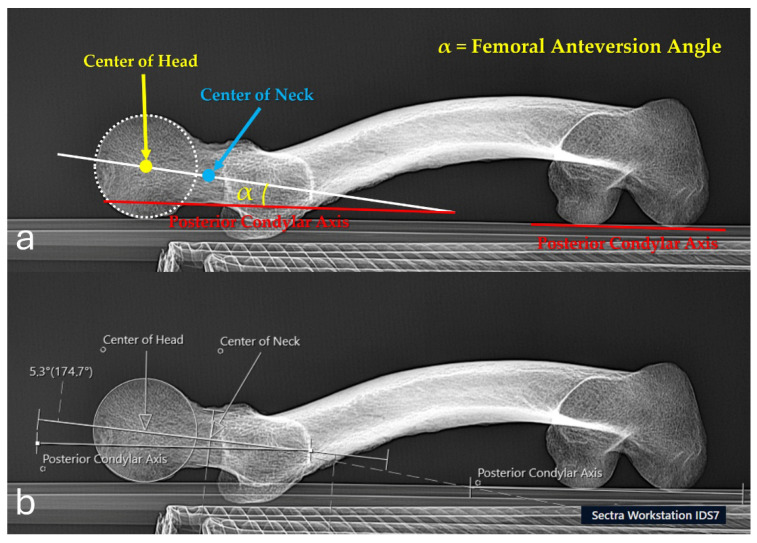
Measurement of femoral neck anteversion on modified Budin radiographs. (**a**) Demonstration of the measurement technique on a modified Budin view: the centers of the femoral head (yellow) and neck (blue) are identified, the line connecting these points represents the femoral head–neck axis, and the posterior condylar axis is drawn along the most posterior aspects of the femoral condyles. The angle α between the femoral head–neck axis and the posterior condylar axis is defined as the femoral neck anteversion angle. (**b**) The same measurement performed on the digital radiography workstation (Sectra IDS 7), showing the angle calculation between the femoral head–neck axis and the posterior condylar axis.

Radiographic measurements were performed using a modified Budin technique adapted for dry bone specimens [11,13]. Each femur was placed on a flat, radiolucent table with the shaft oriented parallel to the table surface, replicating Budin’s original description, in which the femur is aligned horizontally. In this configuration, directing the X-ray beam perpendicular to the shaft reproduces the same relative beam-to-bone geometry as in the seated clinical modified Budin position, where the hip and knee are flexed to 90°. A longitudinal line representing the body midline was drawn on the table surface. The femur was then positioned so that the long axis of the bone formed abduction angles of 20°, 30°, and 40° relative to this midline, corresponding to different simulated “hip abduction” positions. (Figure 4). At each of the three abduction angles (20°, 30°, and 40°), a posteroanterior radiograph (modified Budin view) was obtained, yielding three radiographic images per specimen (Figure 5). All images were acquired with a digital radiography system (Keen Ray DR2800F, Lanmage, Shenzhen, China) and stored in DICOM format for subsequent analysis on the same workstation used for CT measurements (Sectra IDS 7, Sectra AB, Linköping, Sweden). On each modified Budin radiograph, femoral neck anteversion was defined as the angle between the projected femoral head neck axis and the posterior intercondylar line of the distal femur [17], analogous to the CT-based definition (Figure 6).

### 2.5. Reliability of Measurements

Two orthopedic surgeons performed both CT and radiographic measurements. All images were anonymized and presented in random order. The observers were blinded to the specimen identity, to each other’s results, and to the corresponding CT or radiographic values during each measurement session. Each observer measured femoral neck anteversion twice on all CT images and on all modified Budin radiographs at 20°, 30°, and 40° of abduction. The second measurement session was conducted at least 15 days after the first to minimize recall bias. Thus, for each femur and each imaging modality/abduction angle, four values were obtained.

### 2.6. Statistical Analysis

Descriptive statistics were expressed as mean ± standard deviation (SD) and range (minimum–maximum) for continuous variables. For each imaging modality and position (CT, 20°, 30°, and 40° modified Budin views), intra-observer and inter-observer reliability of femoral neck anteversion (FNA) measurements were assessed using intraclass correlation coefficients [ICC (2,1); two–way random-effects model, absolute agreement]. ICC values were interpreted as <0.50 poor, 0.50–0.75 moderate, 0.75–0.90 good, and >0.90 excellent reliability [18]. For subsequent analyses, the mean of the four measurements obtained for each specimen (two observers × two sessions) was used as the representative FNA value for CT and for each abduction angle. Normality of continuous variables and differences between methods were evaluated using the Shapiro–Wilk test, along with visual inspection of histograms and Q–Q plots. The association between CT-derived FNA and radiographic FNA at 20°, 30°, and 40° of hip abduction was examined using Pearson correlation coefficients and simple linear regression, from which correlation coefficients (r) and coefficients of determination (R^2^) were obtained.

Agreement between CT and each modified Budin view was analyzed using the Bland–Altman method. For every abduction angle, the mean difference (bias) and the 95% limits of agreement (LoA = bias ± 1.96 × SD of the differences) between radiographic and CT measurements were calculated and illustrated graphically. In addition, mean absolute error (MAE) and root-mean-square error (RMSE) were computed to quantify the magnitude of measurement error for each abduction angle.

To compare the accuracy of the three radiographic positions, absolute differences between radiographic and CT measurements were analyzed using repeated-measures analysis of variance (ANOVA) when the assumptions of normality were satisfied; if these assumptions were violated, the non-parametric Friedman test was applied. When overall significance was detected, pairwise post hoc comparisons with the Bonferroni adjustment were performed. Overall differences between CT and the three radiographic positions were similarly explored to assess systematic over- or underestimation of FNA. A *p*-value < 0.05 was considered statistically significant. All statistical analyses were performed using IBM SPSS Statistics Base v.23 (IBM Corp., Armonk, NY, USA), and graphs were generated with dedicated graphing software (Microsoft Excel).

## 3. Results

A total of 27 dry femora were included in the analyses. Intra- and inter-observer reliability were excellent across all imaging modalities. For CT, intra-observer ICC (2,1) values were 0.997 (observer A) and 0.995 (observer B), and inter-observer ICCs were 0.994 and 0.995 for the first and second measurement sessions, respectively. For the 20°, 30°, and 40° modified Budin views, intra-observer ICCs ranged from 0.992 to 0.998 and inter-observer ICCs from 0.982 to 0.989, all with narrow 95% confidence intervals within the “excellent” range (Table 1).

**Figure 7:**
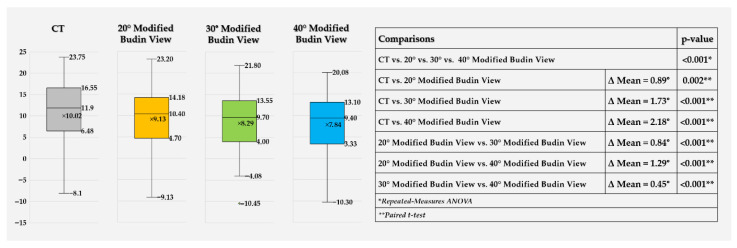
Box-and-whisker plots of femoral neck anteversion (FNA) measured on CT and on 20°, 30°, and 40° modified Budin views. Boxes represent the interquartile range, horizontal lines the median, whiskers the minimum and maximum values, and “×” the mean. The table on the right summarizes overall comparison across all four methods (repeated-measures ANOVA) and pairwise comparisons between methods (paired *t*-tests), presenting mean differences (Δ Mean) and corresponding *p*-values.

Mean CT-derived FNA was 10.02° ± 8.45°. The corresponding mean values on the modified Budin views were 9.13° ± 8.01° at 20°, 8.29° ± 7.79° at 30°, and 7.84° ± 7.50° at 40° of abduction. Repeated-measures ANOVA demonstrated a significant overall effect of measurement method (*p* < 0.001). All modified Budin views yielded significantly lower FNA values than CT, and the degree of underestimation increased progressively as the angle increased from 20° to 40°. In addition, the three radiographic positions differed significantly from each other, with the 20° view producing higher FNA values than the 30° and 40° views, and the 30° view yielding higher values than the 40° view (Figure 7).

**Figure 8:**
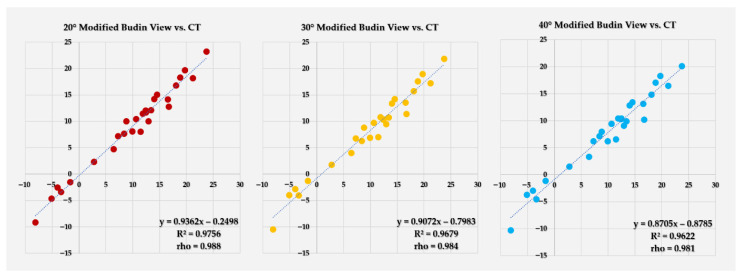
Scatter plots showing the relationship between CT-derived FNA and FNA measured on 20°, 30°, and 40° modified Budin views (left to right). Each point represents one femur (*n* = 27). The dotted line indicates the line of best fit from simple linear regression. The regression equation, coefficient of determination (R^2^), and Pearson correlation coefficient (r) are displayed within each panel.

All modified Budin views showed strong correlation with CT-derived FNA (r = 0.981–0.988), with the highest correlation observed at 20° of abduction (Figure 8).

**Figure 9:**
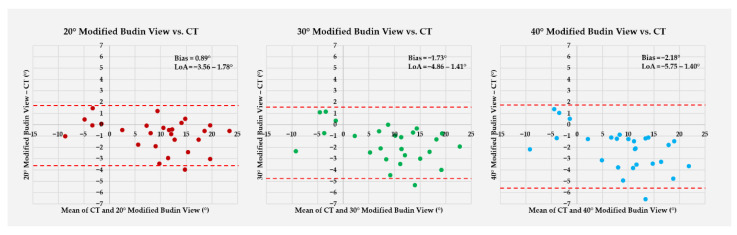
Bland–Altman plots assessing agreement between CT and 20°, 30°, and 40° modified Budin views (left to right). The *y*-axis shows the difference between radiographic and CT measurements (modified Budin view − CT), and the *x*-axis shows the mean of the two methods for each femur. The solid horizontal line represents the mean difference (bias), and the dashed lines represent the 95% limits of agreement (bias ± 1.96 × SD).

Bland–Altman analysis revealed minor but systematic differences between CT and radiographic measurements (Figure 9). All modified Budin views underestimated FNA relative to CT, and both the magnitude of bias and the dispersion of agreement worsened progressively with increasing abduction angle. Error metrics showed the same pattern, with the smallest measurement error at 20° and the largest at 40° (Table 2).

**Figure 10:**
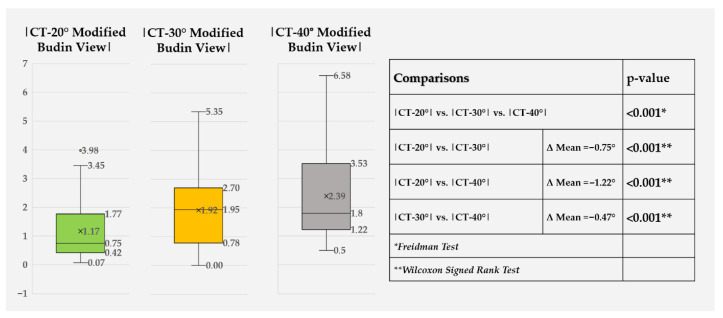
Box-and-whisker plots of absolute error in FNA (|CT − modified Budin view|) for 20°, 30°, and 40° hip abduction angles. Boxes indicate the interquartile range, horizontal lines the median, whiskers the minimum and maximum values, and “×” the mean absolute error. The table on the right summarizes the overall comparison of absolute errors across the three angles (Friedman test) and pairwise comparisons (Wilcoxon signed-rank tests), with mean differences (Δ Mean) and *p*-values.

Absolute error differed significantly among the three radiographic positions (*p* < 0.001) (Figure 10). The 20° view showed the smallest absolute error, followed by the 30° view, while the 40° view showed the largest. Post hoc analysis confirmed significant pairwise differences among all three abduction angles.

## 4. Discussion

In this experimental CT-referenced study of dry femora, hip abduction had a clear, systematic effect on the measurement accuracy of FNA using the modified Budin view. Compared with CT, all radiographic positions slightly underestimated FNA, and this underestimation increased progressively from 20° to 40° of abduction. The 20° modified Budin view produced the smallest bias (−0.89°), the narrowest limits of agreement, and the lowest mean absolute and root-mean-square errors, whereas 40° of abduction was associated with the most significant errors. Absolute differences between CT and radiographic measurements differed significantly among the three positions, with 20° outperforming both 30° and 40°. These findings support the hypothesis that reducing the hip abduction angle improves agreement between modified Budin measurements and CT-derived anteversion. From a geometric standpoint, increasing abduction alters the orientation of the femoral neck relative to both the distal condylar axis and the X-ray beam. As abduction increases, the projected neck axis becomes progressively more oblique to the beam, which may lead to foreshortening of the femoral neck on the radiograph and an apparent reduction in the measured anteversion angle [7,8]. The progressive decrease in the slope of the regression lines and the increasing negative bias with larger abduction angles observed in our data are consistent with this geometric distortion. Conversely, at 20° of abduction, the spatial relationship between the femoral neck, condyles, and X-ray beam appears to approximate more closely the true three-dimensional anteversion, resulting in smaller systematic error and tighter agreement with CT.

The present findings have several practical implications. First, within the boundaries of this controlled experimental model, positioning the femur at approximately 20° of abduction produced the closest agreement with CT and therefore appears to be the most geometrically accurate among the tested modified Budin positions. However, these findings should not be interpreted as direct clinical validation of a 20° protocol in living patients. Rather, they provide experimental evidence that lower abduction angles may improve measurement accuracy under standardized conditions. Whether this advantage can be reproduced in routine clinical practice will require confirmation in future in vivo studies. Second, even in centers where CT is routinely used, radiographic anteversion measurements are often obtained pre- or post-operatively for quick checks or longitudinal follow-up. Using a position associated with minor systematic errors may reduce the risk of misinterpreting clinically relevant changes in femoral anteversion. It may improve comparability between examinations performed at different time points. Finally, our data underscores the importance of precise and reproducible patient positioning during radiographic acquisition; minor deviations in abduction could translate into measurable differences in the recorded anteversion angle. Standardized positioning aids or templates may therefore be helpful when implementing the modified Budin view in everyday practice.

Prior work on Budin-based radiography has consistently shown that a single specially acquired axial-like projection can approximate the CT-derived femoral version [12,19]. Still, virtually all validation studies have treated hip abduction as a fixed, “standard” acquisition parameter [11,13]. The original direct technique described by Budin emphasized obtaining a true axial projection to avoid the pitfalls of indirect calculations. He further noted that near-axial projections obtained with varying abduction may introduce error, particularly in cases of abnormal anteversion [10]. Building on this concept in adults, Lee et al. introduced the modified Budin radiograph—performed in the sitting position with the hip and knee flexed to 90° and typically 30° of abduction—and demonstrated its validity for assessing postoperative femoral stem version relative to CT (r ≈ 0.877), with no significant mean difference [11]. Likewise, Boissonneault et al. applied a similar modified Budin protocol (30° of abduction) to native femora and reported excellent correlation with CT (r ≈ 0.97), along with a small systematic underestimation (mean bias ≈ 0.8°) and limits of agreement of approximately ±6° [12]. Mittal et al. further supported the practicality of this technique in a THA cohort, demonstrating a small, non-significant mean difference from CT (≈0.7°) and high reliability, while also highlighting that most alternative radiographic methods have been validated only to a limited extent across different cohorts. Importantly, across these studies, the abduction angle is largely empirical (commonly 30°) and rarely interrogated as a potential source of systematic measurement error [20].

Our findings extend this literature by demonstrating that hip abduction angle is not a neutral technical detail but a determinant of accuracy when using the modified Budin concept to estimate femoral neck anteversion. Using CT as the reference standard, we observed a stepwise increase in underestimation with increasing abduction from 20° to 40°. Although the radiographic measurements remained strongly associated with CT across all tested positions, correlation alone does not establish agreement or interchangeability. In the present study, the more informative findings were the progressive increase in systematic bias, widening of the limits of agreement, and rise in absolute error with greater abduction. The 20° position produced the smallest bias and tightest limits of agreement (−0.89°; −3.56° to 1.78°), whereas 30° and 40° showed progressively larger negative biases and wider dispersion, with MAE rising from 1.17° (20°) to 1.92° (30°) and 2.39° (40°) (all pairwise comparisons significant). This finding provides important context for interpreting earlier clinical validations, such as those by Boissonneault et al. [12], demonstrating that although high correlation and acceptable agreement can be achieved with a 30° protocol, reducing abduction may further minimize systematic error and improve agreement. This effect may be clinically relevant when small differences in version influence decision-making.

Finally, the broader Budin-incidence literature shows that “modified Budin” positioning is not uniform across applications, underscoring the need for explicit study of abduction. For example, in a different diagnostic context (osteonecrosis), Patrascu et al. described a modified Budin acquisition in which the limb was oriented at a 45° angle to the midplane, and underlined that significant variations in positioning were used in practice [14]. In arthroplasty cohorts, M. Woerner [13] confirmed that a Budin-based view yields better agreement with 3D-CT than an AP-CCD-based method (mean difference ~0.5° vs. ~2.2°) but also reported exclusions due to limited condylar visibility in some patients, again emphasizing that image geometry and projection quality are central to performance. Collectively, these studies align with our conclusion that standardization of acquisition is critical; our contribution is to provide direct evidence that 20° abduction optimizes the modified Budin technique for femoral neck anteversion, offering a concrete, evidence-based refinement to protocols that have historically defaulted to 30° without systematic justification.

This study has several notable strengths. First, it addresses a clearly defined and previously unanswered methodological question by systematically evaluating how different hip abduction angles (20°, 30°, and 40°) influence the accuracy of the modified Budin view, using CT as a reference standard. The experimental design is highly controlled: all measurements were performed on anatomically intact, skeletally mature, dry femora, thereby eliminating confounding factors such as patient motion, soft-tissue overlap, and discomfort with positioning, and allowing the isolated geometric effect of the abduction angle to be assessed. The CT protocol uses a validated three-slice superimposition technique for femoral neck anteversion, providing a robust and widely accepted reference method [15,17,19]. Radiographic acquisition was standardized using a reproducible setup and predefined abduction angles, and all CT and radiographic measurements were performed twice by two blinded orthopedic surgeons, yielding excellent intra- and interobserver reliability across all modalities. Furthermore, the study is statistically well-founded: the sample size was determined a priori based on published variability data and 90% power, and the analysis incorporates complementary agreement and error metrics (ICC, correlation, linear regression, Bland–Altman plots, MAE, RMSE, and repeated-measures comparisons), offering a comprehensive assessment of both reliability and accuracy.

However, several limitations should also be acknowledged when interpreting these findings. The use of dry cadaveric femora, while advantageous for geometric standardization, does not fully replicate clinical conditions in living patients, where soft tissues, pelvic posture, muscle tone, and involuntary motion may alter the effective positioning and radiographic projection; thus, the absolute magnitude of errors observed here may underestimate real-world measurement variability. In addition, practical implementation of the modified Budin view in routine clinical settings may be influenced by factors not present in this experimental model, including restricted hip or knee motion, limited tolerance for the seated position, body habitus, and technical constraints related to radiographic equipment and detector positioning. Therefore, the present findings should be interpreted primarily as defining the geometric accuracy of the technique under controlled conditions, rather than implying that the same positioning can always be reproduced exactly in every patient. Future in vivo studies are needed to determine how these anatomical, functional, and technical constraints affect the feasibility and accuracy of the 20° modified Budin position in daily practice. The sex, age, and pathological status of the specimens were unknown, and this represents an important limitation of the study. The absence of demographic information precluded any subgroup analysis and limits the generalizability of the findings, particularly because femoral morphology and anteversion may vary according to sex, age, and underlying skeletal pathology. Therefore, the present results should be interpreted cautiously when extrapolating to specific clinical populations. The modified Budin technique was adapted for an in vitro setup, and although the angular relationships were carefully modeled, translating a 20° “optimal” abduction angle from a horizontal dry-bone configuration to a seated clinical position may not be perfectly linear; patient comfort and pelvic alignment could impose practical constraints in vivo. In addition, in some patients, particularly those with obesity, increased abdominal girth, or limited sitting tolerance, greater abduction angles may still be required to allow adequate beam passage and detector positioning without soft-tissue obstruction. Therefore, although 20° appeared geometrically optimal under experimental conditions, slightly greater abduction may still be necessary in certain clinical situations. Only three discrete abduction angles were tested, and this represents an important limitation of the study. Therefore, the present data cannot define the full continuous error curve across all possible positions, nor can it rule out the possibility that untested intermediate or lower angles, such as 10° or 25°, might perform as well as or even better than the tested angles. The selected angles were intended to represent clinically practical positions around the commonly used 30° modified Budin protocol rather than to identify a mathematically exact optimum. Future studies should evaluate a broader range of abduction angles to determine whether the relationship between abduction and measurement error is linear or whether another untested position provides the best balance between geometric accuracy and clinical feasibility. The imaging and measurements were performed at a single center using one CT scanner and one digital radiography system by two observers from the same institution, which may limit external validity and fail to fully capture inter-institutional or inter-operator variability. Finally, the study focuses exclusively on geometric accuracy and does not correlate anteversion measurement errors with clinical outcomes or surgical decision-making; therefore, the clinical impact of the observed 1–2° differences between abduction positions must be inferred rather than directly demonstrated.

## 5. Conclusions

When femoral neck anteversion was measured using the modified Budin view, the hip abduction angle had a clear, systematic effect on measurement accuracy. All three radiographic positions (20°, 30°, and 40°) showed excellent reliability and strong linear association with CT; however, method comparison was more meaningfully informed by the agreement analysis, which showed progressive underestimation of anteversion relative to CT, with increasing negative bias, wider limits of agreement, and larger absolute errors at higher abduction angles. Among the tested positions, 20° of hip abduction provided the smallest systematic error, the narrowest limits of agreement, and the lowest mean absolute and root-mean-square errors, indicating the closest agreement with CT-derived anteversion. These findings suggest that, under controlled experimental conditions, a 20° abduction position provides the closest agreement with CT among the tested modified Budin views. However, because this was a dry-bone cadaveric study, the results should be interpreted as geometry-based experimental evidence rather than direct clinical validation. Accordingly, although the 20° position appears promising, its feasibility, reproducibility, and diagnostic performance in living patients must be confirmed in future in vivo studies before routine clinical adoption can be recommended. Future in vivo studies in diverse patient populations are warranted to confirm these experimental results and to evaluate the impact of standardized positioning on clinical decision-making and outcomes.

## Figures and Tables

**Table 1:** Intra- and inter-observer reliability of femoral anteversion measurements on CT and modified Budin views.

Measurement	Comparison	ICC (2,1)	95% CI	Interpretation
CT	Intra-observer reliability			
	Obs. A t_1_ vs. t_2_	0.997	0.993–0.999	Excellent
	Obs. B t_1_ vs. t_2_	0.995	0.989–0.998	Excellent
	Inter-observer reliability			
	Obs. A t_1_ vs. Obs. B t_1_	0.994	0.988–0.997	Excellent
	Obs. A t_2_ vs. Obs. B t_2_	0.995	0.989–0.998	Excellent
20° Modified Budin View	Intra-observer reliability			
	Obs. A t_1_ vs. t_2_	0.997	0.994–0.999	Excellent
	Obs. B t_1_ vs. t_2_	0.992	0.983–0.997	Excellent
	Inter-observer reliability			
	Obs. A t_1_ vs. Obs. B t_1_	0.984	0.965–0.993	Excellent
	Obs. A t_2_ vs. Obs. B t_2_	0.982	0.962–0.992	Excellent
30° Modified Budin View	Intra-observer reliability			
	Obs. A t_1_ vs. t_2_	0.998	0.996–0.999	Excellent
	Obs. B t_1_ vs. t_2_	0.997	0.994–0.999	Excellent
	Inter-observer reliability			
	Obs. A t_1_ vs. Obs. B t_1_	0.983	0.964–0.992	Excellent
	Obs. A t_2_ vs. Obs. B t_2_	0.986	0.970–0.994	Excellent
40° Modified Budin View	Intra-observer reliability			
	Obs. A t_1_ vs. t_2_	0.998	0.995–0.999	Excellent
	Obs. B t_1_ vs. t_2_	0.997	0.993–0.998	Excellent
	Inter-observer reliability			
	Obs. A t_1_ vs. Obs. B t_1_	0.989	0.977–0.995	Excellent
	Obs. A t_2_ vs. Obs. B t_2_	0.987	0.971–0.994	Excellent

Abbreviations—ICC: Intraclass Correlation Coefficient, CI: Confidence Interval, Obs.: Observer, t_1_: First time, t_2_: Second time.

**Table 2:** Agreement between CT and modified Budin views at different hip abduction angles.

Method	r	Δ Mean (X-Ray—CT)	SD of the Difference	95% LoA	MAE	RMSE
20° Modified Budin View	0.988	−0.89°	1.36°	−3.56° to 1.78°	1.17°	1.61°
30° Modified Budin View	0.984	−1.73°	1.60°	−4.86° to 1.41°	1.92°	2.33°
40° Modified Budin View	0.981	−2.18°	1.82°	−5.75° to 1.40°	2.39°	2.82°

Abbreviations—MAE: Mean Absolute Error, RMSE: Root Mean Square Error, LoA: Level of Agreement, SD: Standard Deviation, Δ: Delta (difference).

## Data Availability

The datasets generated and analyzed during the current study are available from the corresponding author on reasonable request. The data are not publicly available due to ethical reasons.

## Abbreviations

The following abbreviations are used in this manuscript:

CT	Computed Tomography
FNA	Femoral Neck Anteversion
ICC	Intraclass Correlation Coefficient
MAE	Mean Absolute Error
RMSE	Root Mean Square Error
LoA	Limits of Agreement
SD	Standard Deviation
ANOVA	Analysis of Variance
PA	Posteroanterior
THA	Total Hip Arthroplasty
